# Interaction between extracellular matrix molecules and microbial pathogens: evidence for the missing link in autoimmunity with rheumatoid arthritis as a disease model

**DOI:** 10.3389/fmicb.2014.00783

**Published:** 2015-01-14

**Authors:** Nidhi Sofat, Robin Wait, Saralili D. Robertson, Deborah L. Baines, Emma H. Baker

**Affiliations:** ^1^Institute of Infection and Immunity, St George’s, University of LondonLondon, UK; ^2^The Kennedy Institute of Rheumatology, Nuffield Department of Orthopaedics, Rheumatology and Musculoskeletal Sciences, University of OxfordOxford, UK

**Keywords:** rheumatoid arthritis, citrullination, lung, periodontal disease, extracellular matrix, infection, microbiome

## Abstract

Rheumatoid arthritis (RA) is an autoimmune disease characterized by inflammation followed by tissue rebuilding or fibrosis. A failure by the body to regulate inflammation effectively is one of the hallmarks of RA. The interaction between the external environment and the human host plays an important role in the development of autoimmunity. In RA, the observation of anti-cyclic citrullinated peptide antibodies (ACPA) to autoantigens is well recognized. Citrullination is a post-translational modification mediated by peptidyl arginine deiminases, which exist in both mammalian and bacterial forms. Previous studies have shown how proteins expressed in the human extracellular matrix (ECM) acquire properties of damage-associated molecular patterns (DAMPs) in RA and include collagens, tenascin-C, and fibronectin (FN). ECM DAMPs can further potentiate tissue damage in RA. Recent work has shown that citrullination in RA occurs at mucosal sites, including the oral cavity and lung. Mucosal sites have been linked with bacterial infection, e.g., periodontal disease, where exogenous pathogens are implicated in the development of autoimmunity via an infectious trigger. Proteases produced at mucosal sites, both by bacteria and the human host, can induce the release of ECM DAMPs, thereby revealing neoepitopes which can be citrullinated and lead to an autoantibody response with further production of ACPA. In this perspectives article, the evidence for the interplay between the ECM and bacteria at human mucosal surfaces, which can become a focus for citrullination and the development of autoimmunity, is explored. Specific examples, with reference to collagen, fibrinogen, and FN, are discussed.

## INTRODUCTION

Rheumatoid arthritis (RA) is an immune-mediated inflammatory disease. It is often associated with chronic disability, early mortality, systemic complications, and places a high socioeconomic burden on society as a whole (McInnes and Schett, 2011). In the last few decades there have been improved treatments for RA, based on immune-modulation of inflammatory pathways. However, up to one-third of people with RA continue to experience high disease activity, despite treatment with strong immunomodulatory drugs such as tumour necrosis factor (TNF) inhibitors, methotrexate, and corticosteroids (Scott et al., 2010). An improved understanding of disease pathophysiology is therefore essential to develop new treatments to address this unmet need.

The development of RA results from a complex interplay between genotype, environment, and lifestyle factors such as smoking (Mahdi et al., 2009). An important clinical aspect in the diagnosis of RA includes the detection of anti-citrullinated peptide antibodies (ACPA) to auto-antigens. Citrullination, also known as demimination, is the conversion of the amino acid arginine in a protein into the amino acid citrulline. Enzymes called peptidylarginine deiminases (PADs) replace the primary ketamine group (=NH) by a ketone group (=O). Citrullination is involved in regulation of development during embryogenesis and in demination-regulated gene expression through histone modifications. Citrulline is not one of the standard 20 amino acids encoded by DNA in the genetic code; it is the result of a post-translational modification. The immune system often attacks citrullinated proteins, thereby leading to autoimmune phenomena in RA.

Twin studies have shown a concordance rate for RA of 15 to 30% among monozygotic twins and 5% among dizygotic twins (13). Genome-wide association analyses have identified immune regulatory factors that may underlie the disease; including PTPN22 among the single nucleotide polymorphisms (SNPs) identified (Wellcome Trust Case Control Consortium, 2007). An association with HLA-DRB1 has been established for RA patients who are positive for rheumatoid factor or ACPA (Gregersen et al., 1987). In keeping with the role of HLA-DRB1 in antigen presentation, a number of studies over the last two decades have shown that auto-reactive immune responses are mediated by T-cell repertoire selection, antigen presentation, or changes in peptide affinity (Panayi, 2006). The shared epitope (SE), carried by the vast majority of RA patients, is a 5-aa sequence motif in the third allelic hypervariable region of the HLA-DRβ chain. Proposed explanations for the link between RA and the SE include molecular mimicry of the SE by microbial proteins, increased T cell senescence induced by SE-containing HLA molecules and a potential pro-inflammatory signaling function that is unrelated to the role of the SE in antigen recognition (Weyand and Goronzy, 1990; De Almeida et al., 2010).

Gene–environment interactions are also important in RA development. Smoking and other environmental risks to the lung, such as silica exposure, increase the risk of RA in people with susceptibility HLA-DR4 alleles (Symmons et al., 1997; Klareskog et al., 2008). Smoking and HLA-DRB1 alleles synergistically increase the risk of developing the anti-citrullinated protein antibodies (ACPA) that are present in the majority of patients with RA (Li et al., 2007). It has therefore been proposed that environmental stress in the lung or other mucosal surfaces may promote post-translational modifications through activation of peptidyl arginine deiminase, type IV (PADIV), which can cause citrullination of mucosal proteins. Loss of tolerance to the neoepitopes generated by citrullination can be detected clinically in people with RA by the ACPA response (Vincent et al., 1999).

For many years, it has been recognized that infectious agents such as *cytomegalovirus, Escherichia coli, Epstein Barr virus, parvovirus,* and *proteus* species may play a role in the development of RA. Recently, the oral pathogen *Porphyromonas gingivalis* has been implicated in the pathogenesis of RA (Mikuls et al., 2014). Products of infectious agents, e.g., heat shock proteins and enzymes responsible for citrullination have been shown in several models to induce immune reactivity. For example, several citrullinated autoantigens can be identified in assays to test for ACPA, keratin, fibrinogen, fibronectin (FN), collagen, and vimentin (van der Woude et al., 2010). Many of the proteins described form part of the extracellular matrix (ECM) common to many structures in the joint, lung, skin, and mucosal tissue. Damage-associated molecular patterns (or DAMPSs) are molecules that can initiate and perpetuate the immune response in the non-infectious inflammatory response. Molecules including fibrinogen and FN, which are abundant in the arthritic joint, have been implicated described as DAMPs in RA pathophysiology and are susceptible to citrullination. It is also possible that cleavage of DAMPs by proteases during the arthritic process may lead to exposure of neoepitopes which are then susceptible to a heightened autoimmune response. Although unifying mechanisms for the link between infection and RA autoimmunity are not entirely established, the theory of molecular mimicry has been proposed (van Heemst et al., 2014). The formation of immune complexes during infection may trigger the induction of rheumatoid factor, which is a high affinity autoantibody against the Fc portion of immunoglobulin, often used in the diagnosis of RA (De Rycke et al., 2004). A link has been described between RA and periodontal disease (PD): *Porphyromonas gingivalis* produces bacterial peptidylarginine deiminase (PAD) which can promote citrullination of mammalian proteins (Wegner et al., 2010). Recently, the gastrointestinal microbiome has also been implicated in the development of autoimmunity (Scher et al., 2012).

## ECM INTERACTIONS IN RA

In the sections below, we discuss the role of common ECM proteins found not only in the arthritic joint, but also highly expressed by mucosal surfaces including the lung, mouth, and gut. We discuss how such ECM proteins may be cleaved and citrullinated at mucosal surfaces, thereby leading potentially to the breakdown of tolerance and the development of autoimmunity in RA.

## COLLAGENS

Collagens comprise a superfamily of ECM proteins which provide a structural framework for many connective tissues. Collagens can be divided into several families or groups based on their exon structure, containing several homologous genes encoding polypeptides that have domains with similar sequences. All collagens have domains with a triple helical conformation (Bella et al., 1994) and are a major constituent of connective tissue. Collagen fibrils composed primarily of type II and XI collagen provide a structural framework to hyaline cartilage (Li et al., 2007), and type I/III and V collagens are a major constituent of skin, tendon, ligaments and bone, demonstrating how the major constituents of the joint require collagen for their structural integrity. Mutations in COL2A1 cause a spectrum of chondrodysplasias, including achondrogenesis II, hypochondrogenesis, spondyloepiphyseal dysplasia, and Kniest and Stickler syndromes (Mundlos and Olsen, 1997). Type II collagen can be injected peripherally to induce RA in murine arthritis in the collagen-induced arthritis (CIA)-model (Williams, 2004), which is one of the most commonly used murine models of inflammatory arthritis.

## FIBRINOGENS

Fibrinogen is a soluble plasma protein. After cleavage by α-thrombin, it is converted to fibrin monomers (Blombäck, 1996). Fibrin monomers self-associate to form an insoluble homopolymeric structure, the fibrin clot. Fibrinogen can also bind to platelets, contributes to the formation of fibrin clots, as well as endothelial cells and leukocytes and plays a multifaceted role in the ECM response to injury. Fibrinogen expression is upregulated at mucosal surfaces during injury, thus participating in inflammatory responses. Congenital lack of fibrinogen results in a bleeding disorder, while increased plasma levels are associated with heightened arterial and venous thrombotic risk (Everse et al., 1998).

## FIBRONECTIN

Fibronectin is an ECM glycoprotein present in tissues and body fluids that is involved in a range of processes, including cellular differentiation, adhesion, migration, wound healing, and neoplastic transformation (Hynes and Yamada, 1982). FN comprises the ECM in joint tissue, including the synovial membranes and cartilage. Expression of FN is upregulated in arthritic diseases including RA and osteoarthritis (Sofat et al., 2012). In addition, FN fragments have been detected in cartilage from people with RA and OA and are responsible for further cartilage matrix degradation (Sofat et al., 2012). Citrullination of FN has been found in RA synovial tissue (Chang et al., 2005) and antibodies to citrullinated FN have been detected in people with RA (Van Beers et al., 2012).

## WHERE COULD CITRULLINATION TAKE PLACE? INSIGHTS FROM MUCOSAL SURFACES

Antibodies to citrullinated peptide antigens are associated with RA and predate disease onset in many cases (Ioan-Facsinay et al., 2008). Since RA autoantibodies often pre-date the development of inflammation in the synovium, it is possible that primary citrullination occurs outside the synovium. It has been suggested that infectious agents release toxins such as lipopolysaccharide (LPS) at mucosal surfaces, triggering an inflammatory response with potential to cause citrullination of ECM. Citrullination may affect ECM proteins found both at mucosal surfaces such as lung, oral and gut mucosa, and in articulating joint tissue, including FN, fibrinogen, and collagen.

## LUNG

Klareskog et al. (2008) suggested that the lung may be a site of citrullination, where co-factors such as smoking and exposure to LPS may result in altered immune status of the lung mucosa. The lung is susceptible to inflammatory responses triggered by infection and autoimmunity (Meyer, 2010). In addition to the increased prevalence of ACPA in smokers (Meyer, 2010) there is also increased ACPA prevalence in RA-related lung disease (Ruiz-Esquide et al., 2012). Respiratory micro-organisms are also linked to the development of RA (Perry et al., 2014).

**FIGURE 1:**
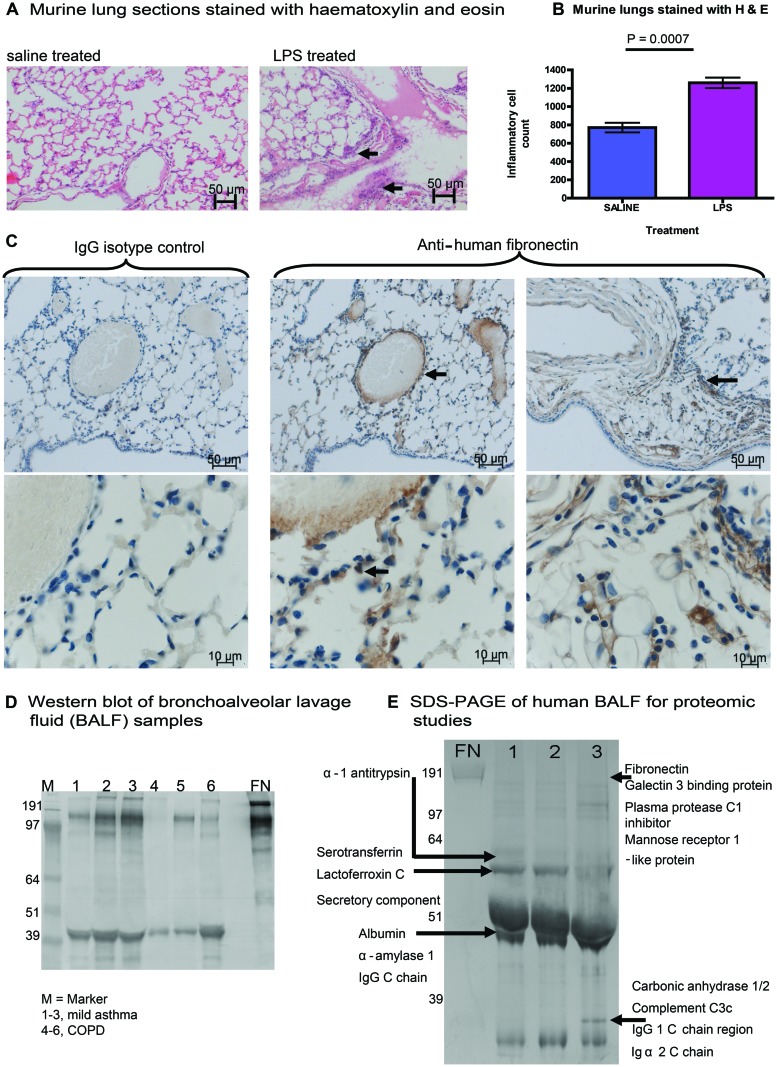
**Expression of fibronectin (FN) in murine and human lung tissue. (A)** Experiments were performed using 6–8 weeks old female BALB/c mice. Mice were anesthetized with isoflurane, then 50 μL of 0.125 mg/kg LPS from *Escherichia coli* serotype 0127:B8 or saline control was administered intranasally. Mice were sacrificed after 24 h: the lungs were dissected out and fixed for 4 h in 4% paraformaldehyde and washed before embedding in paraffin wax. Our murine model showed an inflammatory response to LPS, with oedema, destruction of alveolar architecture and a cellular infiltrate (arrows indicate areas of inflammatory infiltrate). **(B)** The cellular inflammatory infiltrate was quantified in five representative lung fields and was compared between mice treated with LPS or saline control. **(C)** We demonstrated the expression of FN, an ECM molecule in murine lung harvested after LPS treatment. Expression of FN (increased brown staining by horseradish peroxidase) was highly upregulated in LPS treated mice *vs*. saline controls (*n* = 5 in each group). FN protein was detected in the surrounding ECM of alveolar tissue, type II pneumocytes, and the cellular infiltrate (arrows indicate perivascular staining of FN in lung ECM, alveolar tissue, and pneumocytes). For immunohistochemistry, lung tissue was sectioned into 4 μm slices which were stained with haematoxylin and eosin or primary rabbit anti-FN antibody. FN antigen was detected using secondary antibodies conjugated to horseradish peroxidase and analyzed by light microscopy. **(D)** Expression of FN from bronchoalveolar lavage fluid (BALF) is shown in participant samples obtained with informed consent. Samples were obtained from people with mild asthma (1–3) or chronic obstructive pulmonary disease (COPD). SDS-PAGE was performed followed by immunoblotting. The Western blot was treated with a primary antibody to human FN (Sigma), with a secondary anti-rabbit antibody (Sigma) conjugated to alkaline phosphatase for development. **(E)** Bands from samples 1–3 of BALF from participants with mild asthma **(D)** were run separately on SDS-PAGE, bands of interest were cut out and then subjected to analysis using liquid chromatography mass spectrometry (LC-MS). Residues from proteins identified from BALF samples are shown.

To explore the link between inflammation and modification of the ECM matrix in the lung, we investigated the effect of pulmonary LPS exposure on ECM expression in mice. Experiments were performed using 6–8 weeks old female BALB/c mice. Mice were anesthetized with isoflurane (in accordance with UK Home Office regulation), then 50 μL of 0.125 mg/kg LPS from *E. coli* serotype 0127:B8 or saline control was administered intranasally. Mice were sacrificed after 24 h: the lungs were dissected out and fixed for 4 h in 4% paraformaldehyde and washed before embedding in paraffin wax. Our murine model showed an inflammatory response to LPS, with oedema, destruction of alveolar architecture, and a cellular infiltrate (**Figures 1A,B**). For histochemistry, lung tissue was sectioned into 4 μm slices which were stained with haematoxylin and eosin or primary rabbit anti-FN antibody. The expression of FN, an ECM molecule which is expressed in the lung, was highly upregulated in LPS treated mice *vs*. saline controls (*n* = 5 in each group). FN protein was detected in the surrounding ECM of alveolar tissue, type II pneumocytes, and the cellular infiltrate demonstrated by immunostaining with primary anti-FN antibody followed by a secondary antibody conjugated with horseradish peroxidase by light microscopy (**Figure 1C**).

To determine whether the increased FN expression observed in the murine system was also relevant to human lung disease, we investigated the expression of FN in bronchoalveolar lavage fluid (BALF) from people with chronic severe asthma and/or COPD. BALF was probed for FN using SDS-PAGE and Western blotting (**Figure 1D**). We observed expression of full-length FN in all samples tested from human BALF with asthma and COPD. In addition, we observed increased expression of fragmented FN in all samples tested, suggesting cleavage of FN during asthma and COPD. A separate gel was run on SDS-PAGE, and samples of BALF from subjects with mild asthma were analyzed by liquid chromatography mass spectrometry (LC-MS) on 1-D gels by in-gel digestion (**Figure 1E**). Bands from SDS-PAGE were cut from the gel (**Figure 1E**) and subjected to mass spectrometry demonstrating a typical signature of protein expression from BALF samples, including alpha-1 antitrypsin, complement, immunoglobulin, FN was also identified from samples mapping to the cell-binding region and the C-terminal heparin-binding region of FN. These FN regions identified in human BALF are the same regions as have previously been implicated in mediating chronic inflammation in arthritis (Sofat et al., 2012). Taken together, our findings show that acute inflammation in mouse lung induces increased FN expression and that expression and fragmentation of FN can also be demonstrated in human lung BALF extracts.

Other groups have described citrullination of FN in RA (Chang et al., 2005). Upregulation of FN expression at mucosal surfaces including the lung, as we have shown, may consequently contribute to mechanisms of RA pathogenesis such as citrullination. Our data showed increased FN expression in BALF samples and fragmented forms of FN, which may represent fragmentation by proteases. It is possible that FN and/or its fragments mediate chronic inflammation during lung injury and result in citrullination in RA driven by cofactors such as smoking and the SE.

## PERIODONTAL SURFACES

The oral mucosa contains an abundance of bacterial organisms in health and disease. A strong link has been described between PD and RA, giving rise to investigation of the oral microbiome in RA. Recent work has suggested that environmental factors influencing autoimmunity include crosstalk between the human host and oral/intestinal microbiomes. Several lines of investigation have suggested a link between the oral microbes, PD and RA (Wegner et al., 2010; Mikuls et al., 2014). Recent studies have shown that people with RA have a high prevalence of PD. A genome sequencing approach using samples collected from the subgingival biofilm identified a number of organisms, including *Anaeroglobus geminatus, Porphyromonas gingivalis, Prevotella,* and *Leptotrichia* species in people with new-onset RA (Scher et al., 2012).

We investigated the ability of oral microbes to modify ECM proteins. *Porphyromonas gingivalis,* a known pathogen in PD (strain W83 from ATCC) was cultured for 24 h under full anaerobic conditions (3M Concept Plus anaerobic incubator). Bacterial supernatants were extracted and incubated with ECM substrates at 0.5 mg/ml at 37°C, with collection of digestion products from 0 to 180 min after digestion. ECM substrates chosen for these experiments were fibrinogen, FN and type I collagen, as these ECM proteins are found both in arthritic joints and in oral mucosa. The cleavage patterns of ECM substrates were evaluated by SDS-PAGE and Western blotting in the presence and absence of selective protease inhibitors.

**FIGURE 2:**
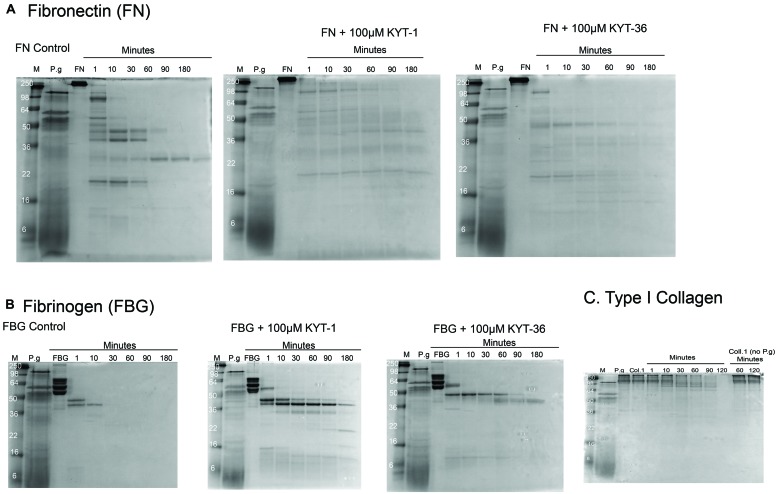
**Cleavage of ECM proteins FN, fibrinogen, and type I collagen by culture supernatants of *Porphyromonas gingivalis.*** Digestion patterns observed over a time course experiment from 0 up to 180 min was performed with culture supernatants from *P. gingivalis* incubated with FN, fibrinogen, and type I collagen, respectively. Digestion patterns for FN **(A)**, fibrinogen **(B)**, and type I collagen **(C)** are shown. For FN **(A)**, almost full cleavage was observed after 180 min, an effect which was delayed in the presence of inhibitors KYT-1 and KYT-36. For fibrinogen **(B)**, full cleavage was observed within 30 min. In contrast, with the gingipain inhibitors, cleavage was not observed in a rapid manner and intermediate cleavage products remained after 180 min of digestion in the presence of KYT inhibitors. For type I collagen, slower cleavage was observed, but was more rapid than without co-culture with *P. gingivalis* supernatants, suggesting that all three ECM molecules tested are cleaved more rapidly in the presence of *P. gingivalis*. Culture supernatants of the anaerobe *P. gingivalis* (strain W83 from ATCC) were produced from 24 h cultures using full anaerobic conditions (3M Concept Plus anaerobic incubator). After culture, bacterial supernatants were extracted and incubated with the substrates at 0.5 mg/ml at 37°C, with collection of digestion products from 0 to 180 min.

We found that culture supernatants from *P. gingivalis* were effective at cleaving all the substrates tested. At 37°C the rate of cleavage was: fibrinogen 30 min for complete cleavage, FN was 180 min for complete cleavage and type I collagen was cleaved at a slower rate over 180 min (**Figure 2**). Intermediate digestion products for all three ECM proteins were demonstrated on SDS-PAGE (**Figure 2**). Our results show that cleavage pattern of ECM protein substrates was distinct for each of the substrates tested. The varying cleavage patterns are likely to have been influenced by the nature of the secreted proteases produced by *P gingivalis*, and that the varying levels of protease expression produced by the microorganism had a differing effect on the digestion pattern and kinetics of specific protein substrates. In the presence of gingipain inhibitors KYT-1 and KYT-36, cleavage of the substrates FN and fibrinogen was strongly inhibited, as demonstrated by the persistence of digestion products in the presence of both protease inhibitors. Due to the likely varying inhibition of selective proteases by specific inhibitors KYT-1 and KYT-36, we observed differing inhibition patterns. However, as observed by the lack of inhibition of complete cleavage of the substrates we tested, it is likely that other proteases are also important in cleaving the substrates tested. Based on our *in vitro* findings, it could be possible that ECM substrates such as fibrinogen, FN, and type I collagen could be cleaved by proteases in the oral mucosa, giving rise to neoepitopes which could then be available for citrullination. Taken together, our findings raise the possibility that modification of ECM proteins in the oral mucosa by bacterial products could drive production of autoantibodies against ECM proteins expressed both in the oral mucosa and in arthritic joints. Inhibition of cleavage of oral ECM proteins may have potential as a new therapeutic target in the management of RA.

## CONCLUDING REMARKS

Our data and reports from other groups demonstrate that mucosal surfaces express ECM DAMPS and that selective proteases can cleave ECM substrates found in the lung and oral mucosa. Our previous work has shown that ECM FN is upregulated in arthritic cartilage (Sofat et al., 2012) and other groups have demonstrated citrullinated FN inhibits apoptosis and promotes production of pro-inflammatory cytokines in RA (Fan et al., 2012). Such findings suggest that ECM proteins shared in the oral mucosa, lung, and the arthritic joint may contribute to the development of autoimmunity in RA. Future work to identify the proteases involved in both cleavage and citrullination of autoantigens, which may then serve to act as DAMPs, both in human and microbial systems, will be crucial to our understanding of disease pathophysiology in RA. Inhibition of cleavage of such substrates may delay the production of ECM DAMPs that are targets for citrullination in RA. Therapeutic strategies aimed at inhibiting such cleavage of ECM substrates may be a novel therapeutic target in RA.

## Conflict of Interest Statement

The authors declare that the research was conducted in the absence of any commercial or financial relationships that could be construed as a potential conflict of interest.
